# Coupled Dynamics in Phenotype and Tissue Spaces Shape the Three-Dimensional Cancer Invasion

**DOI:** 10.1103/prxlife.2.043022

**Published:** 2024-12-24

**Authors:** Austin Naylor, Maximilian Libmann, Izabel Raab, Wouter-Jan Rappel, Bo Sun

**Affiliations:** 1 Department of Physics, Oregon State University, Corvallis, Oregon 97331, USA; 2 Department of Biophysics and Biochemistry, Oregon State University, Corvallis, Oregon 97331, USA; 3 Department of Physics, University of California, San Diego, 9500 Gilman Drive, San Diego, California 10587, USA

## Abstract

The metastasis of solid tumors hinges on cancer cells navigating through complex three-dimensional tissue environments, characterized by mechanical heterogeneity and biological diversity. This process is closely linked to the dynamic migration behavior exhibited by cancer cells, which dictates the invasiveness of tumors. In our study, we investigate tumor spheroids composed of breast cancer cells embedded in three-dimensional (3D) collagen matrices. Through a combination of quantitative experiments, artificial-intelligence-driven image processing, and mathematical modeling, we uncover rapid transitions in cell phenotypes and phenotype-dependent motility among disseminating cells originating from tumor spheroids. Persistent invasion leads to continuous remodeling of the extracellular matrix surrounding the spheroids, altering the landscape of migration phenotypes. Consequently, filopodial cells emerge as the predominant phenotype across diverse extracellular matrix conditions. Our findings unveil the complex mesoscale dynamics of invading tumor spheroids, shedding light on the complex interplay between migration phenotype plasticity, microenvironment remodeling, and cell motility within 3D extracellular matrices.

## I. INTRODUCTION

Three-dimensional (3D) migration of breast cancer cells in tissue scaffolds is far more complicated than the canonical model of cell migration on flat (2D) surfaces has suggested [1–3]. A major complexity is the existence of multiple migration programs that can be activated both internally, through cell mechanotransduction [4,5], and externally, through physical cues from the extracellular matrix (ECM) [6,7]. This leads to the concept of migration phenotype plasticity in cancer cells which was brought to light in a remarkable lesson two decades ago, when clinical trials of drugs that inhibited matrix metalloproteinases (MMPs) failed. At the time, it was commonly believed that MMPs were required for mesenchymal cell migration [8]. However, despite the use of small molecule MMP inhibitors, cancer cells continued to migrate [9,10], as they switched to MMP-independent migration phenotypes [11–13].

We now understand that cancer cells display various migration phenotypes [1,14], such as filopodial (FP) and lamellipodial (LP), which are commonly associated with mesenchymal migration; or actin-enriched pseudopodial (AE) and blebbing (BB) cells, which execute amoeboidal migration programs. All of the four phenotypes have characteristic morphologies, morphodynamics, and mechanical interactions with the ECM [7,15]. In previous studies, we observed that individual breast cancer cells exhibited the ability to transition between various migration phenotypes as they navigated through the three-dimensional ECM [16,17]. However, a fundamental question still remains unanswered: How do these transitions in migration phenotype contribute to the invasive behavior of solid tumors, which comprise hundreds to thousands of cells?

In this study, we examine tumor spheroids formed by MDA-MB-231 breast cancer cells which invade into surrounding ECM consisting of type-I collagen. We use deep-learning algorithms to automatically segment all the disseminated cells from confocal imaging stacks [18,19], and we apply previously trained machine-learning models to classify the cell migration phenotypes based on their morphology [17]. Combining quantitative experiments and theoretical modeling, we demonstrate that the closely coupled phenotype dynamics and migration characteristics of invading cancer cells play key roles in determining the invasive potential. By systematically varying the mechanical properties of the ECM, we show consistent features in mesoscale dynamics under broad conditions. Our results highlight the plasticity of cancer cell migration programs and underscore the effect of ECM remodeling in reciprocally regulating cancer invasion.

## II. RESULTS

### A. Experimental characterization of mesoscale dynamics of invading cancer cells

**FIG. 1.**
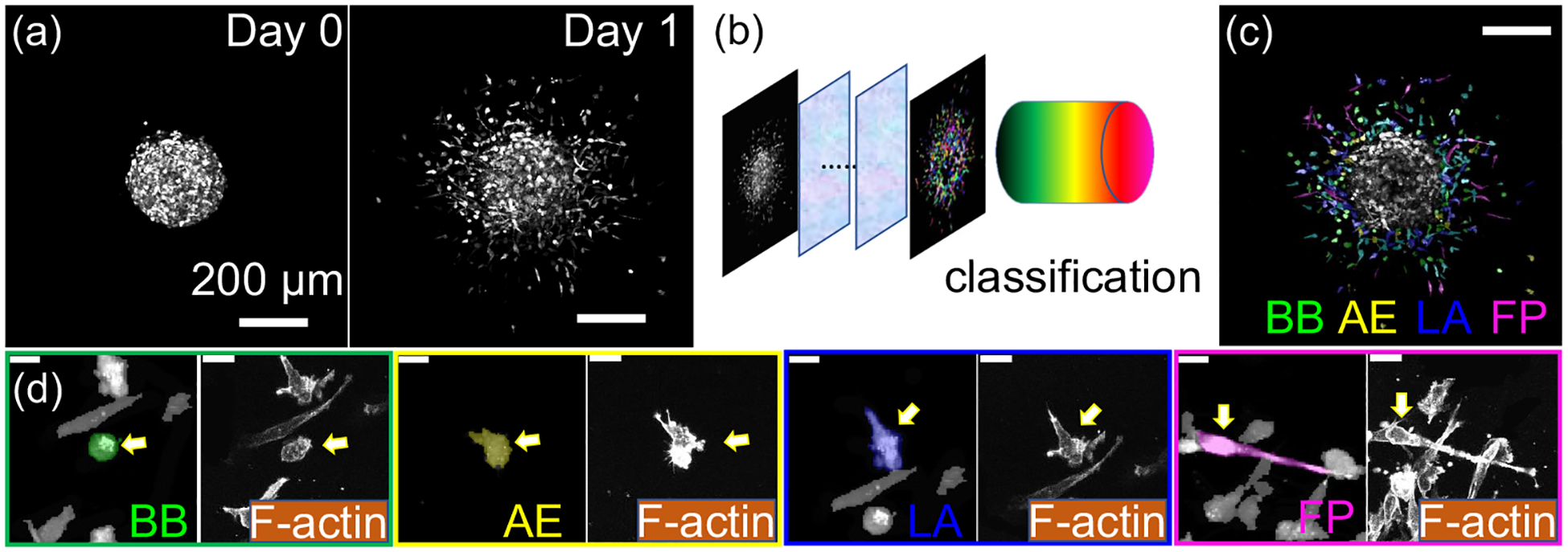
Artificial-intelligence-assisted segmentation and classification of breast cancer cells disseminating from a tumor spheroid. (a) Snapshots of a tumor spheroid consisting of GFP-labeled MDA-MB-231 cells invading a 3D extracellular matrix (ECM). Images are 2D projections of 3D confocal stacks. The spheroid is embedded in type-I collagen ECM with a concentration of 1.5 mg/ml. (b) Employing the Projection Enhancement Network (PEN [19]) paired with the CellPose algorithm [18], cells are automatically segmented to obtain cell masks. Based on the masks, cells are classified into four migration phenotypes using a previously reported machine-learning model [17]. (c) Disseminated breast cancer cells from the spheroid in panel (a) are automatically segmented and classified. Note that we focus on cells that have already separated from the tumor spheroids. Here we render the image in panel (a) with cell masks whose color represents one of four migration phenotypes. BB, blebbing; AE, actin-enriched pseudopodial; LA, lamellipodial; and FP, filopodial. Scale bar: 200 μm. (d) Zoom-in views show typical cell morphology and actin cytoskeleton structure corresponding to each migration phenotype. Scale bars: 20 μm.

In order to characterize the invasion dynamics of cancer cells, we embed spheroids formed by MDA-MB-231 breast cancer cells in 3D ECM consisting of type-I collagen (see Supplemental Material (SM) [20], which includes Refs. [21–24], for additional information about the experimental methods and analysis). MDA-MB-231 is a triple-negative line of invasive breast cancer cell widely used in cancer research. Each spheroid is continuously imaged for 24 h at a frame rate of 15 min using a laser scanning confocal microscope (Leica SPE). In a typical experiment, several hundreds of cells separate from the spheroid and invade the collagen matrix within 24 h, while the spheroid expands less than 10% of its initial radius of approximately 200 μm [Fig. 1(a)].

In order to minimize the photon damage, confocal slices are set to be 10 μm apart, ten times the resolution in the *x*-*y* plane. Recently we have developed PEN (Projection Enhancement Network), a deep-learning algorithm that segments cells from 3D image stacks with low axial resolution to create accurate 2D projections of cells [19] (see also SM [20], Sec. S2). After obtaining the cell mask, we then use our previously reported machine-learning model to classify a cell into one of four migration phenotypes [17]: BB, blebbing; AE, actin-enriched pseudopodial; LA, lamellipodial; and FP, filopodial [Figs. 1(b) and 1(c); see also SM, [20] Sec. S2]. Due to high cell density within the spheroid, cells cannot be reliably segmented until they have disassociated from the spheroid body. Therefore, in this study we focus on the cells that have already separated from tumor spheroids. Figure 1(c) shows a snapshot where the disseminating cells are color-coded outside of a tumor spheroid represented in gray scale.

These migration phenotypes have distinct morphological characteristics. For instance, the cortical pressure of a BB cell continuously drives the formation of rounded blebs at the cell membrane [25–27]. AE cells, on the other hand, demonstrate elevated actin polymerization that drives sharp protrusions [1,13]. The FP cells consist of distinguishable F-actin bundles extending across the polarized cell body [28,29], while the LA cells feature fan-shaped leading edges of migration [30,31].

These morphological features allow us to classify a cell based on the geometry of its 2D projection at close to 90% accuracy [17]. Indeed, we find the different phenotypes also show characteristic actin cytoskeleton structures that correspond to distinct migration programs of cancer cells in 3D ECM [7,15].

**FIG. 2.**
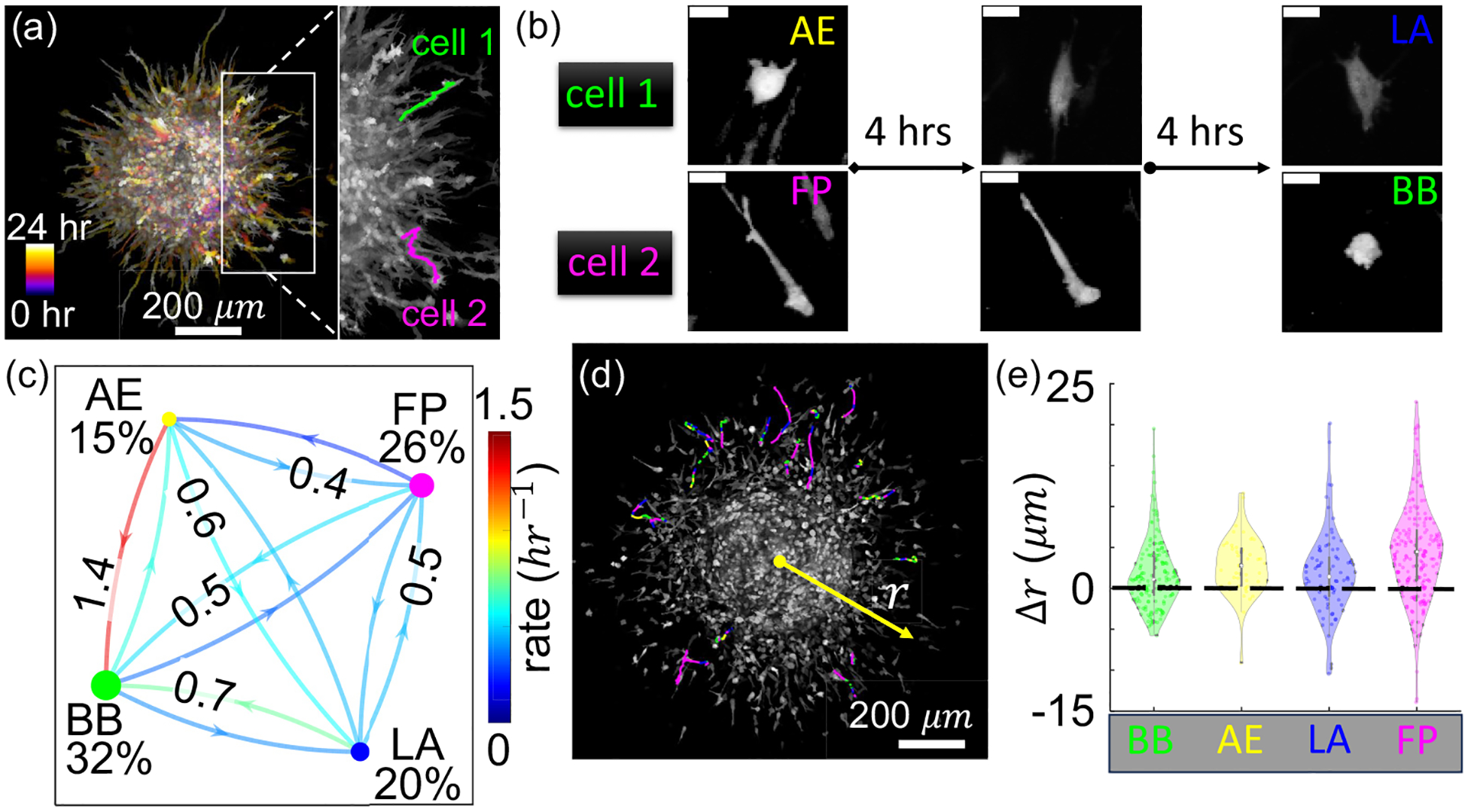
Breast cancer cells disseminating from a spheroid in 3D collagen ECM exhibit migration phenotype transitions. (a) A temporal projection showing the invasion paths of MDA-MB-231 cells from a tumor spheroid where two representative cells are highlighted. (b) Snapshots showing two cells [highlighted in panel (a)] make phenotype transitions during invasion. Scale bars: 20 μm. (c) The transition matrix quantifies the phenotype dynamics of disseminating MDA-MB-231 cells from a tumor spheroid embedded in type-I collagen ECM with a concentration of 1.5 mg/ml. Percentage next to the nodes indicate the fraction of each phenotype. Cells in the intermediate state, which consists about 6% of the population, are not shown. Numbers along the line-arrows show transition rates—probability of making a particular transition per unit time in units of h^−1^. Colors of the lines are linearly mapped to the transition rates. Colors of the nodes represent the corresponding phenotype, and the sizes of the nodes are proportional to the phenotype fraction. More than 7000 events (including dwell events) over 24 h of invasion are sampled in computing the transition rates as well as phenotype fractions. See SM, Sec. S3 [20], for additional examples of transition matrices from repeated experiments. (d) To accurately follow cells for a longer period of time (at least 10 h), 23 cells are manually tracked and their trajectories are color-coded by instantaneous phenotype. (e) Histograms showing the radial step size distributions associated with each phenotype using the trajectories obtained in panel (d). The steps are displacements in the radial direction over frame intervals of 15 min. Phenotype abbreviations and representing colors in panels (b)–(e): BB, blebbing (green); AE, actin-enriched pseudopodial (yellow); LA, lamellipodial (blue); and FP, filopodial (magenta). The mean ± standard deviation of the step sizes are as follows: BB, (1.7 ± 4.2) μm; AE, (2.8 ± 3.5) μm; LA, (1.8 ± 5.2) μm; and FP, (4.5 ± 5.9) μm.

We find cells disseminating into the surrounding ECM demonstrate both intercellular heterogeneity and single-cell plasticity in migration phenotypes. Figure 2(a) shows the temporal projection of cells from a tumor spheroid invading the ECM consisting of 1.5 mg/ml type-I collagen. We highlight two typical cells which made phenotype transitions as they continuously disseminated. Note that the transitions are not limited within the amoeboid (between blebbing and actin-enriched pseudopodial) or mesenchymal (between lamellipodial and filopodial) modes. The frequent switching between amoeboid and mesenchymal models highlights the dynamic nature of breast cancer cells’ migration phenotype plasticity.

To quantify the migration phenotype plasticity, we compute the transition matrix by sampling the mesoscale dynamics exhibited by all cells after they have disseminated from the spheroid. Figure 2(c) shows the transition rates as well as the fraction of each phenotype obtained by sampling all events during 24 h of invasion. In all our repeated tests (see SM [20], Sec. S3, for additional experimental results), we find that BB cells appear most frequently (accounting for 1*/*3 of the population), while actin-enriched pseudopodial (AE) is the least common (accounting for 1*/*6 of the population). Cells rapidly switch between all migration phenotypes, with a characteristic rate of one transition every 2 h. We note that the rate imbalance between BB and other phenotypes favors the accumulation of blebbing cells, which explains its highest fraction in the population.

We find that the motility of cancer cells depends on the migration phenotype. To illustrate this, we manually track cells that exhibit high invasiveness whose trajectory can be followed for at least 12 h [Fig. 2(d)]. Autocorrelation analysis shows the cell motility has no long-term memory (see SM, Sec. S3 [20]) and, therefore, can be considered as a random walk given our temporal resolution. We calculate the cell’s radial step size Δ*r*, which is the displacement in the radial direction over 15-min intervals, and then we compare the distributions of these step sizes based on the cells’ initial phenotypes. As shown in Fig. 2(e), it is evident that filopodial cells make the largest outward steps (Δ*r* > 0), which drive the overall invasion. On the other hand blebbing cells make nearly equal outward and inward moves and, therefore, contributing little to the metastatic dissemination of the tumor spheroid. Together, the results suggest that filopodial cells may actively lead the invasion process.

**FIG. 3.**
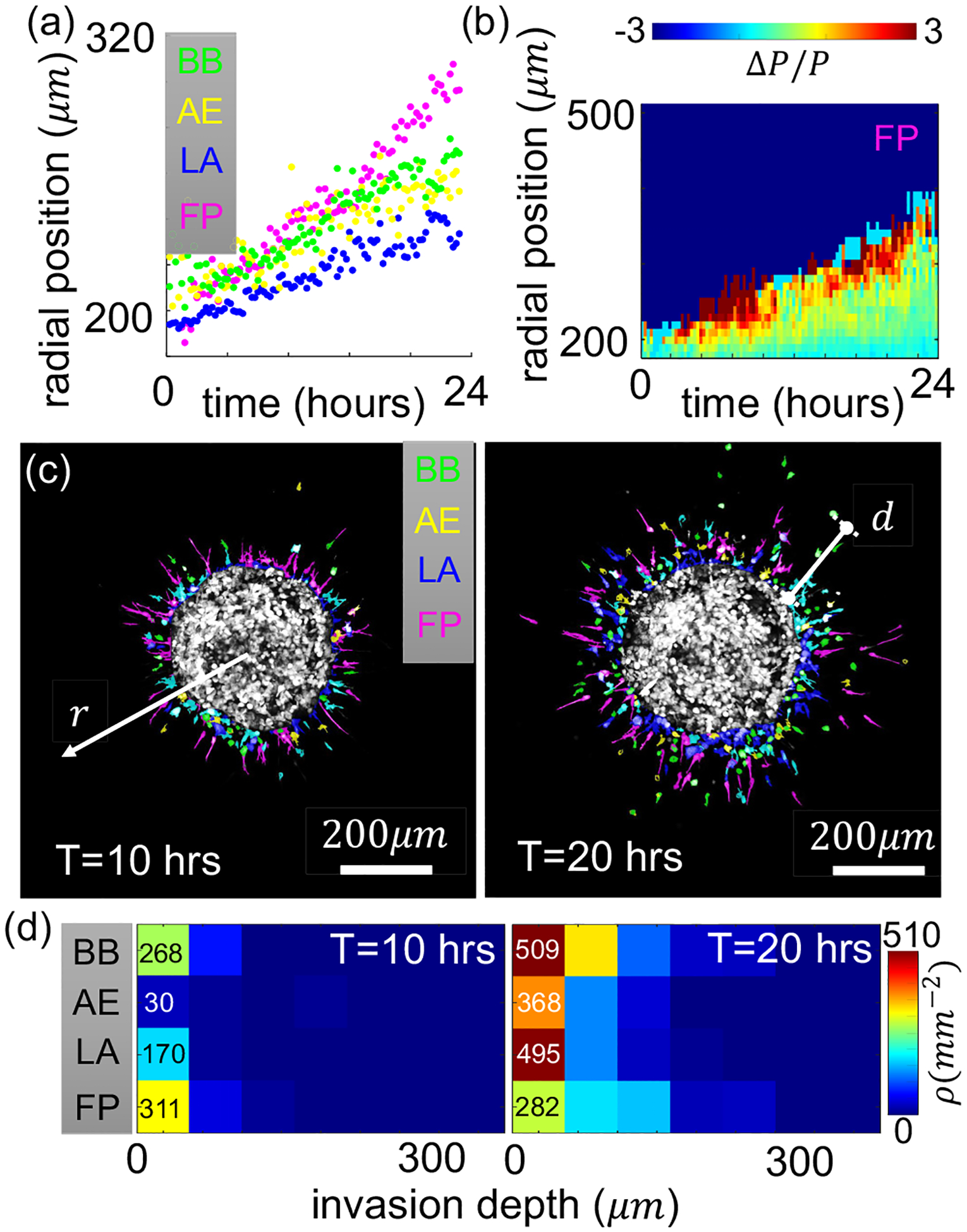
Spatial-temporal phenotype dynamics of breast cancer cells disseminating from a spheroid in 3D collagen ECM. (a) The average radial position of cells associated with each phenotype over 24 h of invasion from a spheroid consists of MDA-MB-231 cells. The spheroid radius is approximately 200 μm and is embedded in type-I collagen ECM of concentration 1.5 mg/ml. Time zero is empirically determined when cells have dissociated from the spheroid. (b) Relative difference (Δ*P*) between the instantaneous local filopodial (FP) fraction *P*(**r***, t*) and the mean filopodial (FP) fraction *P* (over recorded time and field of view). Here we take advantage of the rotational symmetry and compute $\frac{\Delta P}{P}$ by only considering radial positions. (c), (d) Phenotype composition evolves as a spheroid invades the 3D ECM. (c) Snapshots after *T* = 10 h and *T* = 20 h of invasion. Color of the cells represents the migration phenotype classified via the machine-learning model. (d) Heat maps show the density *ρ* of cells of each phenotype at varying invasion depth at *T* = 10 h and *T* = 20 h. Here the invasion depth *d r – r*_*s*_ is defined as the distance to the spheroid boundary and *r*_*s*_ is the spheroid radius [see schematics in panel (c)]. For spatially resolved cell density, we calculate $\rho_{i}\frac{N(r_{i}⩽r⩽r_{i+1})}{\pi (r_{i+1}^{2}-r_{i}^{2})}$, where *r*_*i*_ are bound radial position bins of width 50 μm and *N*(*r*_*i*_ ⩽ *r* ⩽ *r*_*i*+1_) is the number of cells whose 2D projection falls within the *i*th radial position bin. Phenotype abbreviations in panels (a)–(e): BB, blebbing; AE, actin-enriched pseudopodial; LAm lamellipodial; FP, filopodial.

In order to further establish the filopodial cells as the leading phenotype during tumor spheroid invasion, we calculate the average instantaneous radial position of each phenotype. As the example shown in Fig. 3(a), filopodial cells consistently have the largest radial position while lamellipodial cells lag behind. After 24 h, the average radial position of filopodial cells have moved 120 μm from the tumor boundary, which is twice the amount for the advancement of lamellipodial cells.

Not only do the filopodial cells exhibit a larger radial displacement, they also show a spatial distribution pattern with strong enrichment at the invasion front. Figure 3(b) shows the instantaneous local fraction of filopodial cells *P*(*r, t*) in comparison with their global proportion *P*, where $\frac{\Delta P}{P}=\frac{\left|P(r,t)-P\right|}{P}$. Here *P*(*r, t*) is obtained by counting the fraction of filopodial cells within the spatial annulus with an inner radius of *r* and an outer radius of *r* + 50 μm, and at time *t*. *P* is obtained by counting all disseminated cells over the entire 24 h of recording. It is clear that filopodial cells are particularly more frequent near the invasion front. The result shows a stark increase of the filopodial cell fraction by as much as 300% at the invasion front compared with the bulk average.

To gain further insight into the spatial-temporal distribution of cell migration phenotypes during 3D invasion, we compute the cell density within the annuli surrounding the tumor spheroid. For convenience, we define the invasion depth *d* as shown in Fig. 3(c) to be the distance to the tumor boundary. We find that cells in the BB, AE, and LA phenotypes are accumulating near the tumor boundary, with proportions close to their bulk average. However, filopodial cells first accumulate and then disperse to lower densities near the tumor boundary [Fig. 3(d)]. These observations suggest that filopodial cells may exhibit spatial-temporally evolving transition dynamics deviating from their bulk averaged rates. Specifically, enrichment at the invasion front and depletion near the tumor boundary have increased the average radial position of filopodial cells as shown in Fig. 3(a).

### B. Mesoscale dynamics of tumor spheroid invasion under varying ECM conditions

It is known that the mechanics and structure of ECM play important roles in modulating 3D cancer invasion. Therefore, we have also examined MDA-MB-231 tumor spheroid invasion in ECM with varying physical properties. To this end, we embed the spheroids in matrices made of regular, as well as methacrylated, type-I collagen. The latter can be photo-crosslinked via a low dose of UV light [32] (see SM [20], Sec. S4, for more details).

**FIG. 4.**
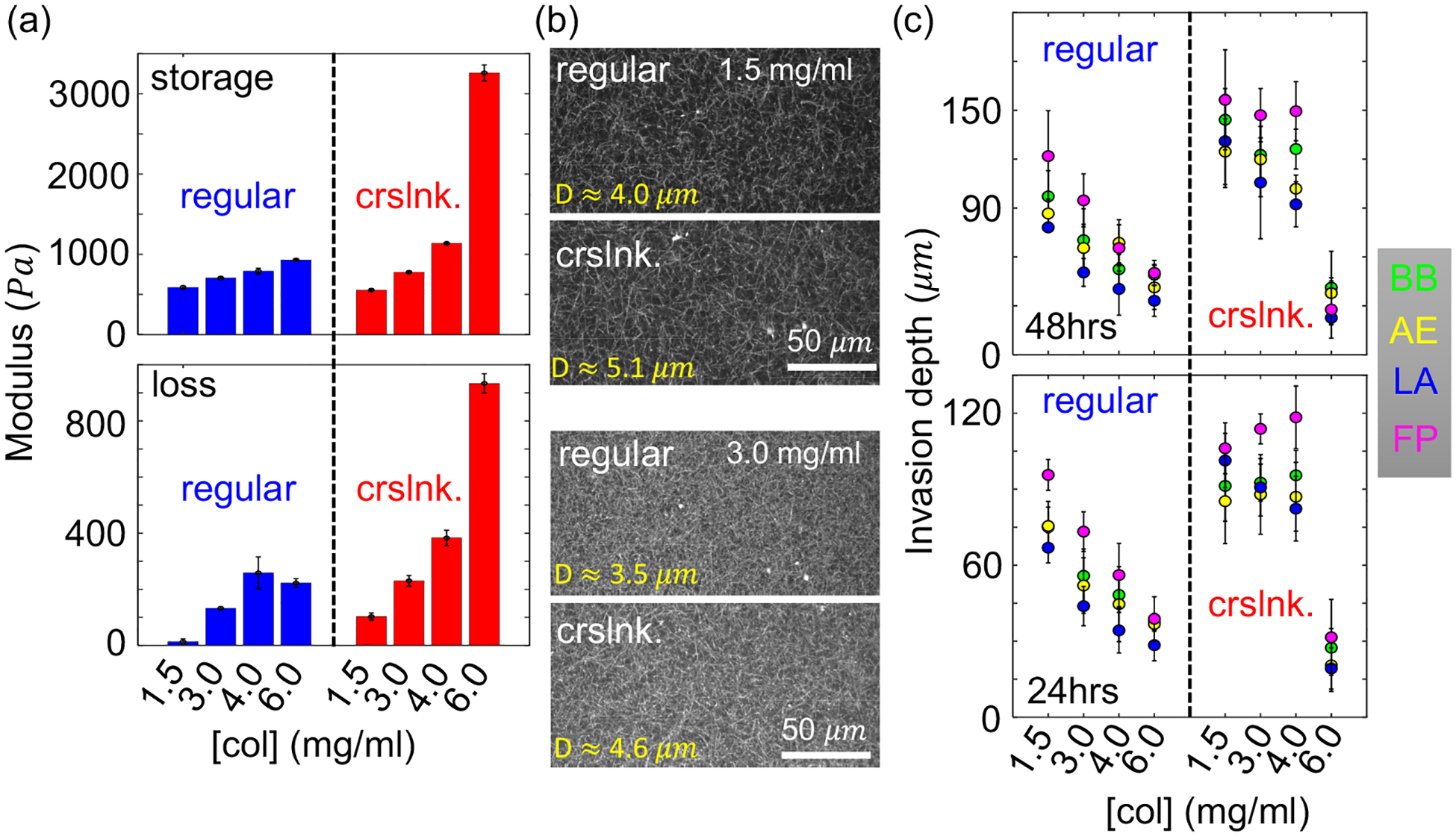
Filopodial cells (FP) lead the invasion of tumor spheroid under various ECM mechanical conditions. (a) The storage and loss moduli of regular collagen ECM and photo-crosslinked (abbreviated as crslnk.) collagen ECM at varying concentrations [col]. (b) Sample confocal reflection images of regular and photo-crosslinked collagen ECM at concentrations of 1.5 mg/ml (top) and 3.0 mg/ml (bottom). The pore sizes *D* are computed from the diameters of max-fitting disks after binarizing the images. See SM [20], Sec. S4, for more details. (c) Average invasion depths of each phenotype disseminated from MDA-MB-231 tumor spheroids into ECM of varying conditions. Top: Invasion depth after 2 days. Bottom: Invasion depth after 1 day. Abbreviations in panels (a)–(c): crslnk., photo-crosslinked; BB, blebbing; AE, actin-enriched pseudopodial; LA, lamellipodial; and FP, filopodial. The error bars are means and standard deviations from four to five spheroids for each ECM condition.

We vary the collagen concentration from 1.5 to 6 mg/ml and find that higher concentration generally increases the stiffness and viscosity of the ECM. Figure 4 shows that photo-crosslinked (abbreviated as crslnk.) matrices have significantly larger storage and loss moduli when the collagen concentration is greater than 3 mg/ml. It is also noted that the stiffness does not increase by crosslinking at the lowest concentration of 1.5 mg/ml.

The ECM microstructure, such as the pore size, modulates cancer cell migration by controlling the physical barriers. Combining confocal reflection imaging and automated image processing, we find the pore size of the ECM decreases as collagen concentration increases [Fig. 4(b)], which is consistent with previous reports [33]. On the other hand, for the same collagen concentration, we find photo-crosslinked ECM generally have larger pore sizes [Fig. 4(b)], although at larger collagen concentrations ([col] > 4 mg/ml), the pore size cannot be reliably determined from optical images.

We have examined the spheroids disseminating at varying ECM conditions. In particular, we image the samples after 24 h and 48 h of invasion and apply our artificial intelligence models to quantify the migration phenotypes of all cells outside of the spheroids. Figure 4(c) shows the average invasion depths of each phenotype. Similar to the result shown in Fig. 3(a), filopodial cells are consistently the leading phenotype in all cases, except at the highest collagen concentration ([col] = 6 mg/ml), where MDA-MB-231 are strongly confined by the ECM and barely separate from the spheroids. It is interesting to note that the invasion depth is curtailed by higher concentration of regular collagen, but not for photo-crosslinked collagen in the ECM. We suspect there is a critical pore size, *D*_*c*_, below which the invasion is strongly suppressed. Conversely, when the ECM pore size is greater than *D*_*c*_, invasion is mainly controlled by ECM stiffness, which facilitates cell motility by promoting adhesion and traction force generation [34]. Since photo-crosslinked ECM has larger pore size as well as rheological moduli, the combined effect would provide a putative explanation of the observed correlation between invasiveness, collagen concentration, and crosslinking.

### C. Modeling the mesoscale dynamics of cancer invasion

**FIG. 5.**
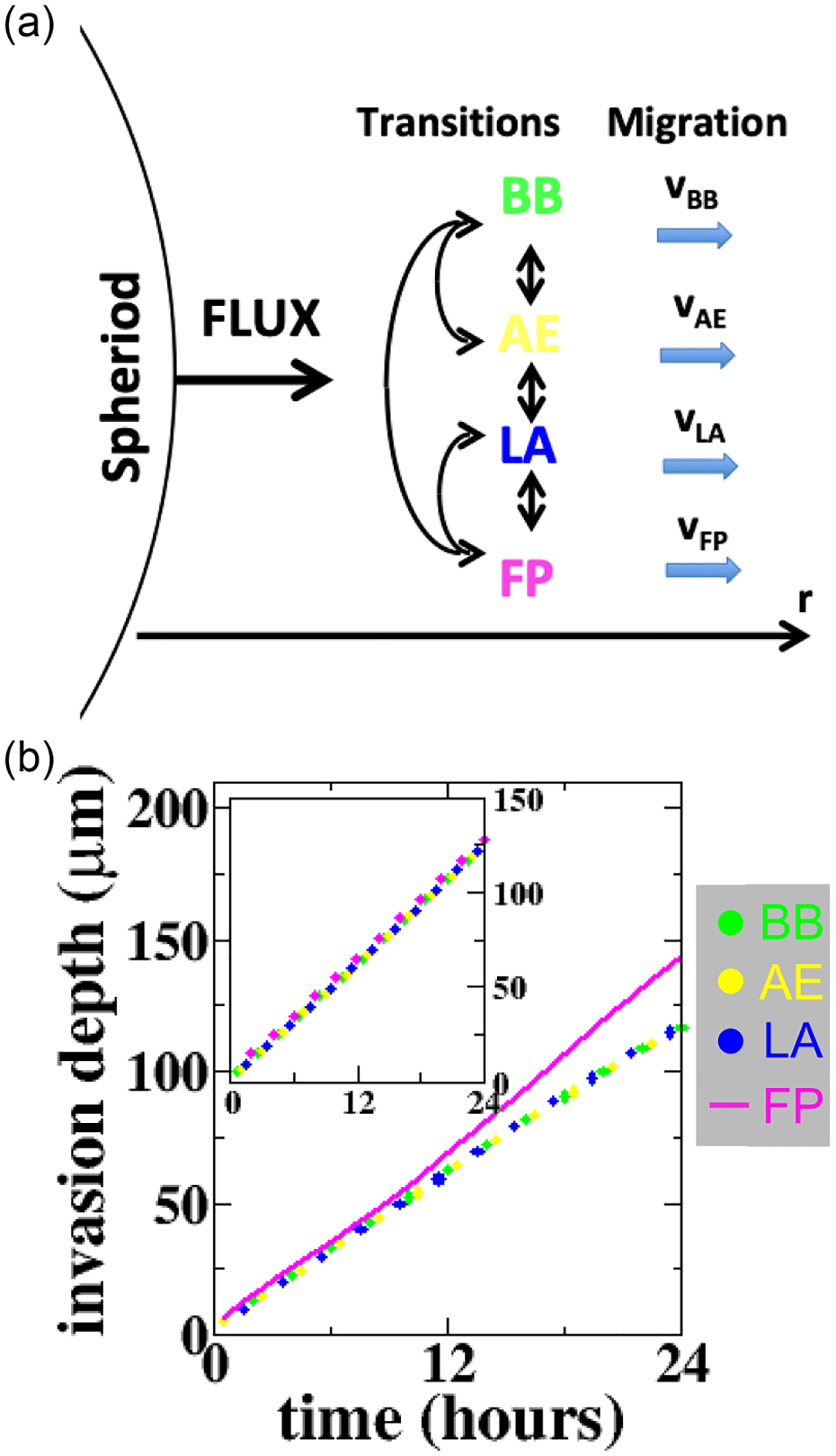
Computational modeling of the cancer invasion mesoscale dynamics (a) Schematic illustration of the computational model. Density of the four phenotypes are solved along a line extending from the spheroid. Each phenotype is introduced into the computational domain through a flux at the boundary of the spheroid and migrates in the positive *r* with fixed speed. The transitions between phenotypes are computed using the rates given in Fig. 2(c). (b) Invasion profiles of each phenotype from the computational model. The inset shows the average invasion depth as a function of time for constant transition rates. The main panel shows the same quantity but for the case that the transition rates from phenotype FP close to the spheroid are linearly decreased over time [see Eq. (3)].

In order to better understand the role of mesoscale dynamics in cancer invasion, we have constructed a simple computational model inspired by the experiments. We are particularly interested in explaining the larger average invasion depth of the FP cells compared to the other phenotypes. For simplicity, our model considers the cell density profiles along a line starting from the spheroid and extending away from it, as schematically shown in Fig. 5. For each phenotype, the following equations model the transition between the different phenotypes as well as the migration away from the spheroid. For phenotype AE, the equation reads

(1) $$\frac{d[\text{AE}]}{dt}=r_{\text{BB}\to \text{AE}}[\text{BB}]+r_{\text{FP}\to \text{AE}}[\text{FP}]+r_{\text{LA}\to \text{AE}}[\text{LA}]-(r_{\text{AE}\to \text{BB}}+r_{\text{AE}\to \text{FP}}+r_{\text{AE}\to \text{LA}})[\text{AE}]-H(-\nabla [\text{AE}])v_{\text{AE}}\nabla [AE].$$

Here, the first three terms describe the transitions between other phenotypes to AE and the fourth term encodes the transition from AE to all other phenotypes. The last term encapsulates the movement of the cells belonging to this phenotype and is multiplied with the Heaviside function so that, consistent with experiments, cells always move away from the organoid. Similar equations can be written down for the other phenotypes, resulting in four coupled equations.

At the edge of the organoid, we assume a constant flux *F* of cells into the space, taken to be equal for all phenotypes: *D***∇**[*X*] = *F*, where *X* stands for BB, AE, LA, or FP. The rates appearing in the equations are given by the experimental data [Fig. 2(c)], while the speed of the cells [υ_AE_ in Eq. (1), similar for other phenotypes] is taken to be the average as determined in Fig. 2(e). The tabulated simulation parameters are included in the SM, Sec. S5 [20].

We first examine whether the larger steps of FP cells compared to the other phenotypes could explain the observed invasion depth of FP cells. For this, we solve the equations with constant rates and determine the mean invasion depth as a function of time. Since the system equilibrates rapidly, however, the difference in speed does not result in a marked difference in average invasion depth. This is shown as an inset in Fig. 5, where the four phenotypes have the same invasion depth as a function of time.

Inspired by the experimental results, which demonstrate that the number of FP cells close to the spheroid drops significantly after approximately 6 h, we next adjust the transition rates of all phenotypes to FP. Specifically, we assume that for cells within 50 μm of the tumor, these rates are linearly decreasing, starting at *t* = 8 h and reaching 0 at*t* = 24 h. Thus, the new transition rate of the *X* phenotype to FP, ${\tilde{r}}_{X\to \text{FP}}$, obeys:

(2) $$\begin{array}{ll} {\tilde{r}}_{X\to \text{FP}}=r_{X\to \text{FP}}, & t \end{array}⩽8\;\text{h},$$

(3) $$\begin{array}{ll} {\tilde{r}}_{X\to \text{FP}}=\left(\frac{24-t}{18}\right)r_{X\to \text{FP}}, & t \end{array}>8\;\text{h},$$

where *X* stands for AE, BB, and LA. In other words, we assume that as cells continuously invade the ECM, the transition rates to the FP phenotype approach zero near the tumor boundary. The invasion profile of this simulation is presented in Fig. 5(b), which indeed shows filopodial cells leading the invasion compared with other phenotypes. The simulation confirms that complete mesoscale dynamics, which accounts for the cell migration phenotype transitions, is necessary to understand the spatial-temporal profiles of disseminating tumor cells. We should note that adding a diffusion term to the equations, with a diffusion constant determined from the radial step size distributions in Fig. 2(e), does not change our qualitative results (see SM [20], Fig. S14).

To further validate the assumption of adjusted transition rates near the tumor spheroids, we use simulations to compute the time-resolved enrichment factors associated with each phenotype within 50 μm of the tumor boundary. The enrichment factor is the ratio of transition rates into versus out of a phenotype, which decreases over time for filopodial cells. The simulation results are supported by experimental measurements, for spheroids in both regular and photo-crosslinked ECM (see SM [20], Sec. S4).

### D. Mesoscale dynamics of invading tumor spheroids may be modulated by ECM remodeling

**FIG. 6.**
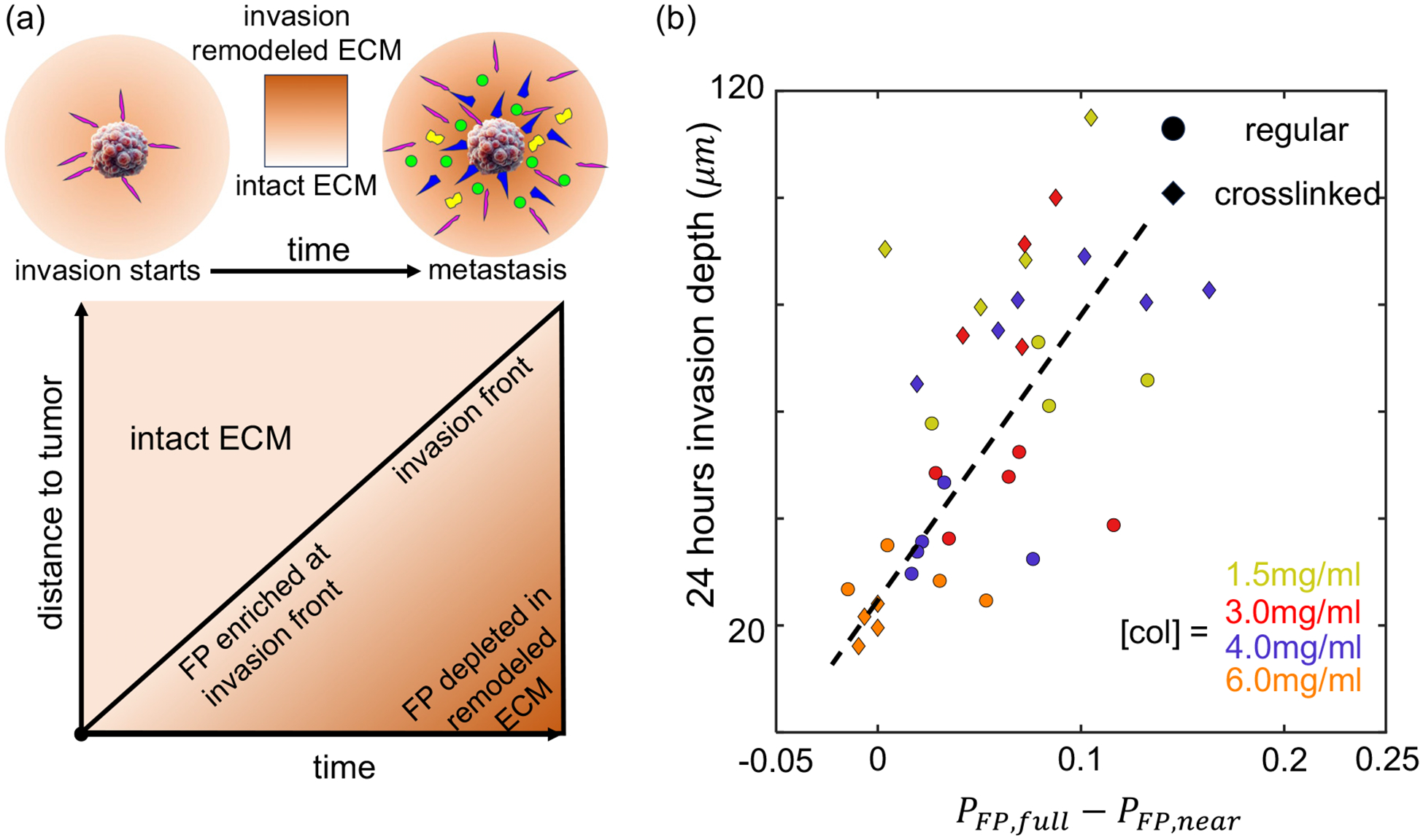
ECM remodeling by disseminated cancer cells may regulate the migration phenotype transitions and thereby the metastatic potential of breast tumors. (a) Schematics of the putative mechanism where the tumor invasion front separates remodeled ECM and intact ECM. The level of ECM remodeling is stronger near the tumor boundary. Filopodial (FP) enrichment factor is negatively correlated with the level of ECM remodeling, thus enriching the FP phenotype near the invasion front and depleting the FP phenotype near the tumor boundary. (b) Difference between the FP fraction of the full field of view (*P*_FP,full_) and the FP fraction within 50 μm of the spheroid boundary (*P*_FP,near_) positively correlate with the invasion depth. Here each data point represents one MDA-MB-231 spheroid in 3D collagen ECM. Colors represent ECM collagen concentration. Dots symbols: Regular collagen ECM. Diamond symbols: Photo-crosslinked collagen ECM. Dashed lines: Linear fit of all data points as a guide to the eye.

Through a combination of experimental and mathematical analysis, we demonstrate that 3D tumor invasion operates as a layered stochastic process characterized by phenotype dynamics that evolve over time and by phenotype-dependent cell motility. The results suggest that filopodial cells are favored at the invasion front to penetrate intact ECM, and they are increasingly inhibited near the tumor boundary as the invasion persists. A plausible explanation for the phenomena is the ECM remodeling by invading cells [Fig. 6(a)].

At the invasion front, filopodial cells extend long, fingerlike protrusions. Compared with characteristic protrusions of other phenotypes, filopodia encounter the least resistance against the physical constraints of the 3D extracellular matrix [35]. Intact ECM fibers may also support cell adhesion that promotes cell polarization and actin polymerization into mature F-actin bundles [36,37]. On the other hand, the ECM may be weakened by invading cells, as confirmed by our direct measurement of ECM storage modulus over invasion process (see SM [20], Sec. S4). Previously we employed holographic optical tweezers to show that metastatic breast cancer cells disseminated from breast tumors softened the ECM along their invasion paths by secreting matrix metalloproteinases (MMPs) and mechanical forces [38]. Due to the plasticity of the ECM [39,40], the effect of ECM remodeling by hundreds of cells may accumulate over time, creating an evolving mechanical microenvironment surrounding the tumor spheroids. Such alterations lead to more porous and compliant ECM, which could transform the landscape of migration phenotypes. In particular, the remodeled ECM will reduce the likelihood of transitions towards the filopodial state far behind the invasion front.

The association of ECM remodeling with altered migration phenotype dynamics suggests that, further behind the invasion front, filopodial cell type is suppressed. Specifically the local fraction of FP cells near the tumor boundary *P*_FP,near_ will deviate from the bulk fraction of FP cells *P*_FP,full_. *P*_FP,full_ − *P*_FP,near_ is expected to be positively correlated with the invasion depth, which is a proxy of distance between the invasion front and the tumor boundary. This is indeed the case as shown in Fig. 6(b). Except for the highest collagen concentration (6 mg/ml) where cells barely disseminate from the tumor spheroids, *P*_FP,full_ − *P*_FP,near_ grows with invasion depth for both regular and photo-crosslinked collagen ECM.

## III. DISCUSSION AND CONCLUSION

Migration phenotype plasticity is of fundamental importance in the metastasis of solid tumors. While traditionally thought to be a rather static status of cancer cells, we now know that cells make rapid, spontaneous transitions between different migration phenotypes. Switching of phenotype and cell motility concurrently happen at the same time scale, leading to the mesoscale dynamics that controls the invasion process. In this study, we examine the mesoscale dynamics exhibited by breast cancer cells disseminating from tumor spheroids into surrounding 3D collagen matrices. Leveraging image analysis by artificial intelligence, we segment, classify, and track MDA-MB-231 cancer cells from confocal image stacks [Figs. 1(a)–1(c)]. Despite the large number of cells disseminating from each spheroid, the accuracy of cell detection and classification is comparable with our previous work where cancer cells were sparsely seeded in the ECM [17]. Indeed, the machine-determined cell state agrees well with the characteristic actin cytoskeleton organization of the corresponding migration phenotype [Fig. 1(d)].

By following the mesoscale dynamics of hundreds of cells simultaneously, we collect large datasets to quantify the phenotype dynamics as transition matrices, and we quantify cell motility by their step size distributions (Fig. 2). Similar to the case of isolated MDA-MB-231 cells, the actin-enriched pseudopodial (AE) state serves as a hub state mediating mesenchymal to amoeboid transitions, thanks to the large AE to BB transition rate. Quantitatively the transition matrices differ for cells uniformly dispersed in ECM and cells disseminating from spheroids. Indeed, the invasion process is accompanied with dramatic ECM remodeling [38], leading to a nonstationary, and spatially heterogeneous, microenvironment that impacts the phenotype dynamics.

We notice that each phenotype is associated with distinct motility characteristics. In particular, filopodial (FP) cells have a larger step size moving away from the spheroid and therefore drive the dissemination the most. FP cells are also enriched at the invasion front and depleted near the spheroid boundary (Fig. 3). These observations strongly suggest that FP cells are leaders in the invasion process. Interestingly, recent studies show that the blebbing state boosts the survival of cancer cells [41]. Therefore, tumors may take advantage of the mesoscale dynamics to enhance the overall metastatic potential.

We systematically varied the ECM physical property and found that filopodial cells are consistently the leading phenotype (Fig. 4). Increasing collagen concentration stiffens the ECM and reduces ECM pore size, which strongly suppresses spheroid invasion. Conversely, crosslinking the collagen fibers also stiffens the ECM but enlarges the ECM pore size, therefore restoring the invasiveness of tumor spheroids. Except for spheroids in the highest collagen concentration (6 mg/ml), where cells barely disseminate, filopodial cells have the largest average invasion depth compared with other phenotypes, underscoring their role as the leader cells during 3D tumor invasion.

The experimental results show that 3D cancer invasion is a layered stochastic process where phenotype switching is coupled with phenotype-dependent motility. Importantly, using mathematical models we show that the phenotype-dependent step sizes alone are not sufficient to explain the leader behavior of filopodial cells, precisely because of the rapid phenotype transitions [Fig. 5(b), inset]. Instead, as motivated by the experimental observations, it is necessary to drop the assumption of equilibrium phenotype dynamics. Our mathematical model shows that making transitions toward the FP state gradually smaller near the tumor boundary can establish filopodial as the leading phenotype [Fig. 5(b)]. This assumption is consistent with experimental observations (see SM [20], Sec. S4).

Our findings reveal remarkable time-evolving mesoscale dynamics when cancer cells disseminate from a solid tumor. At the invasion front, the physical barrier and confinement imposed by the intact extracellular matrix (ECM) influence the transition of cell phenotypes, favoring the enrichment of filopodial phenotype. As the invasion front progresses outward from the tumor, it leaves behind an ECM that is subject to ongoing remodeling by disseminating cells. The ECM remodeling accumulates due to its plasticity, resulting in a mechanically weakened matrix that is less supportive for filopodial cells. Consequently, phenotype dynamics does not reach equilibrium. Instead, filopodial cells are enriched at the invasion front and increasingly depleted near the tumor boundary [Fig. 6(a)]. Experiments with a wide range of ECM conditions support the prediction, such that the level of nonequilibrium phenotype dynamics, manifested as a reduction of filopodial cells near the tumor boundary, is an accurate predictor of invasion depth [Fig. 6(b)]. We note that plastic remodeling of ECM is implied in a wide range of cancer types [42]. It is therefore imperative for future research to examine the interplay between ECM remodeling and mesoscale cell dynamics as a potential oncogenic factor in other solid tumors.

In this study we use spheroid invasion as an *in vitro* model that recapitulates many key physiological factors of metastatic breast cancer. It will be interesting to examine if primary tumors demonstrate similar mesoscale dynamics. It is also very worthwhile for future study to investigate the molecular mechanism that mediates the change in migration phenotype dynamics by ECM remodeling. The results may provide valuable therapeutic insights into breast carcinoma and other metastatic solid tumors.

## IV. METHODS

Additional details of experimental setup, data analysis, and computational modeling can be found in the Supplemental Material, Secs. S1–S5 [20].

Raw data can be found at Figshare [43,44]. The software for cell segmentation and classification can be found at GitHub [45,46].

## Supplementary Material

SI
