# Supplementary material for: Coupled Dynamics in Phenotype and Tissue Spaces Shape the Three-Dimensional Cancer Invasion
Source: PRX Life. Author manuscript; Available in PMC 2025 Aug 22. (PMC12369984; doi:10.1103/prxlife.2.043022)
Supplement: SI [file NIHMS2092068-supplement-SI.pdf]

Supplementary Information

S1. Additional Details of the Experimental Setup

Cell Culture

We obtain GFP-labeled MDA-MB-231 human breast carcinoma cells (GenTarget) and maintain them according to the manufacturer's instructions. Briefly, growth media is prepared using Dulbecco's Modified Eagle Medium (Gibco) supplemented with 10% Fetal Bovine Serum (Gibco), 1% 100x Penicillin-Streptomycin (Gibco), and 0.1 mM Non-Essential Amino Acid (ThermoFisher). Cells are kept in culture flasks in a tissue culture incubator at 37°C and 5% CO_2.

3D Spheroid Formation

3D spheroids are formed using GFP-labeled MDA-MB-231 human breast carcinoma cells cultured as described above. Spheroids are formed by seeding cells into a V-bottom, 96-well plate with a cell repellent surface (Greiner Bio-One). Specifically, $100\mu L$ of 100 cells/μL cell solution is gently pipetted into each well of the 96-well plate, for a total of 10,000 cells per well, and then the plate is centrifuged at $290g$ for 3 minutes. The plate is then placed in a tissue culture incubator at 37°C and 5% CO_2. 24 hours later, $100\mu L$ of 6 μg/mL collagen in growth medium (Corning; 9 - 11 mg/mL) is gently pipetted into each well before centrifuging the plate at $100g$ for 3 minutes. The plate is then placed back in a tissue culture incubator. The spheroids will be ready to use anywhere between 3 and 6 days after this step. For consistency, all spheroids were used 5 days after the addition of collagen to each well.

Embedding 3D Spheroids in 3D Collagen Gels

We prepare two different types of collagen gels at 4 different concentrations for a total of 8 different gel conditions. We prepare gels with and without crosslinking at concentrations of 1.5, 3, 4, and 6 mg/mL with pH 7.4. Collagen gels without crosslinking are prepared from high-concentration rat tail collagen 1 in acetic acid (Corning; 9 - 11 mg/mL). We dilute the collagen with DMEM growth medium, 10x PBS, and 0.1M NaOH to the desired final concentration. Collagen gels with crosslinking are prepared from photocrosslinkable collagen (Advanced Biomatrix, PhotoCol). We dissolve the lyophilized collagen sponge in acetic acid to a base concentration of 8 mg/mL to then dilute further for gel creation. We dilute the collagen with DMEM growth medium, 10x PBS, 0.1M NaOH, and 1% by volume photoinitiator to the desired final concentration. We mix and keep all solutions at 4°C prior to the gelation process at 37°C. We place the neutralized collagen solution in a glass-bottom dish (MATTEK) before carefully pipetting a 3D spheroid into the liquid solution and mixing. We allow the collagen solution to gel at 37°C for 30 minutes to fully polymerize. Finally, the gels are placed under a 365nm UV-light for 5 minutes to induce photocrosslinking. For consistency, all gels are placed under the UV-light regardless

of its ability to crosslink.

Microscopy and Image Processing

Continuous imaging is done with a Leica TCS SPE laser scanning confocal microscope (10x air objective, NA=0.4) and equipped with a stage-top incubator (Ibidi). Experiments last for 24 hours with images captured at a rate of 1 frame per 15 minutes. Raw images are grayscale z-stacks where x-y planes are imaged every 10μm for a total of 140μm stack size. Each individual image has a resolution of 1024 $\times$ 1024 pixel^2. Snapshots are taken with the same settings, but are taken only at 0 hours, 24 hours, and 48 hours rather than continuous imaging. These Z-stacks are grayscale images that will eventually be passed into our deep learning model for cell segmentation. There is a chance that some spheroids may be touching the bottom of the dish, which may cause some cells to move along the glass rather than invading into the ECM. To ensure our data is of only cells invading into the ECM, we cut the bottom 2 slices (20 μm) from any Z-stack that has cells touching the bottom of the dish.

Immunofluorescence

In order to image the F-actin of our cells, we first need to stain the F-actin using a standard immunofluorescence staining protocol (ThermoFisher), image-iT Fixation/Permeabilization Kit (Invitrogen), and Alexa Fluor 488 Phalloidin (Invitrogen). First, after all media is removed from the sample, 4% formaldehyde solution in PBS is added to the sample and incubated for 15 minutes at room temperature to fix the sample. After washing the sample 3 times with PBS for 5 minutes each wash, 0.5% Triton X-100 in DPBS, a permeabilization agent, is added to the sample and incubated for 15 minutes at room temperature. After washing the sample another 3 times with PBS for 5 minutes each wash, 3% BSA blocking solution, to prevent non-specific binding, is added to the sample and incubated for 60 minutes at room temperature. Then, after the blocking solution is removed, the F-actin Phalloidin stain is added to the sample and incubated for 20 minutes at room temperature. The sample is then washed 3 times with PBS for 5 minutes each wash, and is finally ready to be imaged. We image the F-actin on the Leica TCS SPE laser scanning confocal microscope. To ensure a high-resolution image of the F-actin, we use a 20x oil objective (NA=0.6), take an x-y plane image every 2μm, and take a 3 frame average across the images.

Cell tracking

As detailed in section S2, the confocal images are processed through out deep-learning pipeline to obtain a mask for each cell in each image frame. Once a cell mask is detected, we use the maximum inscribed circle (MIC) to estimate the center of the cell (see [1] for more details). Previously, we have shown that MIC agrees very well with direct nucleus staining when determining the cell position.

For most cells, the deviation is less than 10% of the cell long axis. The root mean squared deviation is approximately 3 microns[1]. After cell centroids are obtained, we either employ manual or automatic tracking. For manual tracking that allows us to obtain long trajectories, we use the ManualTrack plugin of ImageJ. For automatic tracking, we apply the Crocker-Grier algorithm [2] to establish 2-frame trajectories. The analysis is done for all consecutive frames in order to obtain the transition events. We limit the automatic tracking to 2-frames because after testing a number of available algorithms we find manual tracking still produces the most reliable long trajectories. We have also verified that 2-frame trajectories by automatic tracking are consistent with manual tracking results.

S2. Additional Details of Cell Segmentation and Phenotype Classification

Deep Learning Used for Cell Segmentation

Since we are interested in the morphodynamics of invading cells from the spheroid, we need to be able to create masks of each individual cell for each frame. As manually drawing masks for hundreds of cells is not an efficient process, we employ the use of deep learning to create masks for the individual cells. Specifically, we use the projection-enhancement network (PEN)[3] our lab has previously developed on top of CellPose[4]. Figure S1 below shows the architecture of PEN and the proposed workflow associated with using PEN. Briefly, a Z-stack 3D image is passed as input to PEN, which is then operated on by 5 different scales of convolutional blocks. The outputs of each branch are then stacked together and operated on by a final convolutional block to produce a single RGB image of equal horizontal and vertical resolution as the input. This output image is an RGB image where axial Z information from the 3D image stack is stored as color, rather than the raw grayscale image stack. We have compressed a 3D image stack to a single 2D image without losing the axial Z information, which has been stored as color in the RGB image. Now, we can pass this 2D RGB image into our 2D segmentation network of choice, CellPose, and get a greater accuracy in segmentation. PEN is a crucial addition to our segmentation procedure, as our spheroids have many cells that overlap axially, causing standard 2D segmentation networks to struggle. By encoding the axial data in an RGB image that is passed to the 2D network, the network is better able to segment cells that are overlapping, as the network now knows where in the stack the overlapping cells belong.

Our PEN plus CellPose model was evaluated on a low-density cell dataset (N = 4082) and a high-density cell dataset (N = 111). The high-density cell dataset is a crucial addition to the training of the network, as many of our spheroids have areas of high-density cells that are overlapping axially. We use the following metrics to evaluate our model: Jaccard Index, Precision, Recall, and Quality. For the low-density cell dataset, our model has the following metric values: 0.523, 0.574, 0.854, and 0.807, respectively. For the high-density cell dataset, our model has the following metric values: 0.518, 0.616, 0.766, and 0.782, respectively. For more information on our model, please refer to our previously published paper [3].

Machine Learning Used for Phenotype Classification

Once we have segmented our cells, we next need to classify those cells into their respective phenotype so that we can analyze the morphodynamics of the invading cells. To do this, we employ the use of Support Vector Machine Learning (SVM) [5, 6]. From the masks created by our cell segmentation, we first calculate 21 geometric measures which collectively quantify the shape of a cell. These geometric measures characterize cell size (such as area and perimeter), deviation from circle (such as aspect ratio and form factor), surface topology (such

as solidity), and backbone curvature (such as curl) [1]. For our MDA-MB-231 cells in 3D collagen matrices, there are four distinct phenotypes: blebbing (BB), actin-enriched pseudopodial (AE), lamellipodial (LA), and filopodial (FP). Figure S2 below shows an example cell from each of the 4 phenotypes. With 3766 manually labeled single cell images we have trained a supported vector machine (SVM) to calculate probability scores for a cell to belong to each morphological phenotype. We assign a cell to the phenotype if the probability is greater than 60%. For a small fraction of cells ($\approx 10\%$), none of the 4 phenotypes have a probability greater than 60%, so we deem these cells to be in an intermediate state (IM). After training the model on the 3766 image training dataset, we create a new dataset for validation. This unseen dataset was prepared with 50 cell images per phenotype, and the model performs at 88% accuracy on this unseen dataset. For more information on our model, please refer to our previously published paper [1].

Characteristics of breast cancer cell migration phenotypes

The table below summarizes the shape, signaling and cell-ECM interactions of four different migration phenotypes seen in breast cancer. Other solid tumors cells may demonstrate similar phenotypes.

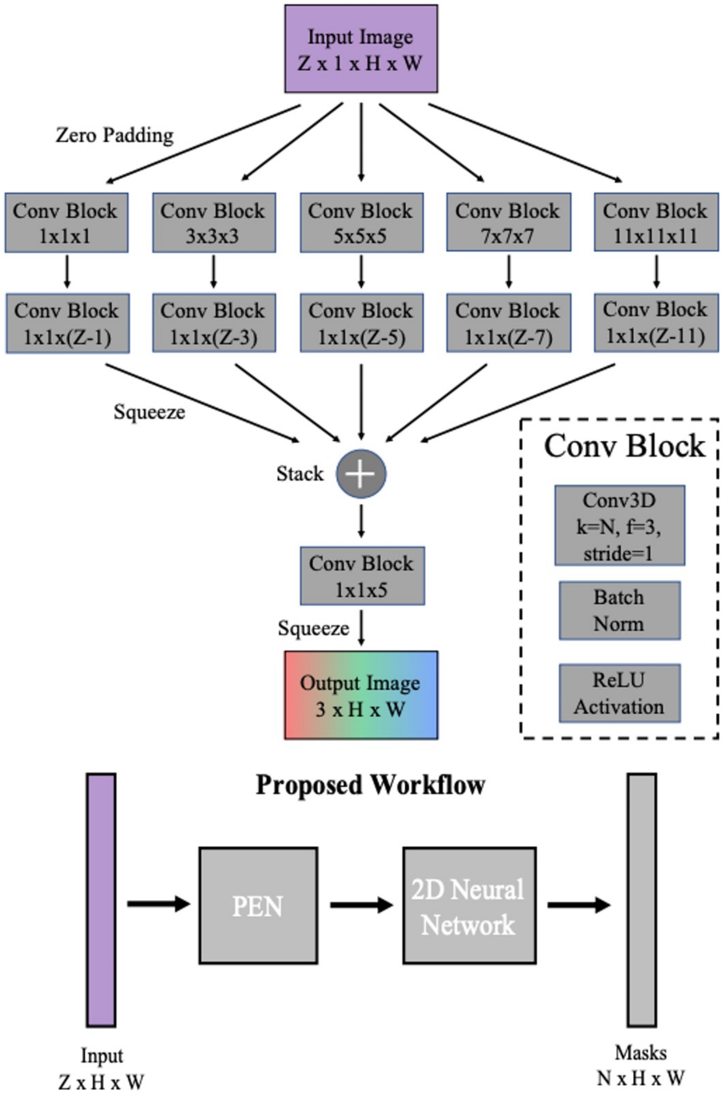

Figure S1: PEN Architecture and proposed workflow. PEN will work with any 2D neural network of choice that allows 2D RGB images as input. Our 2D neural network of choice is CellPose.

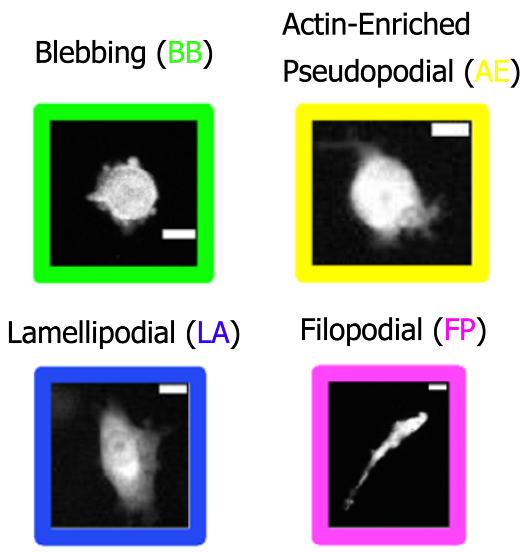

Figure S2: An example cell from each of the 4 phenotypes. Scale bars are all 20 μm.

	Amoeboidal vs Mesenchymal	Morphology Features	Signaling Features	Interaction with ECM	References
**Blebbing** 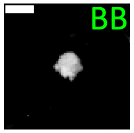	Amoeboidal	Rounded cells that have smaller, rounded protrusions on the membrane	Cortical stress drives the formation of rounded blebs at the cell membrane. No clear polarization of cytoskeleton and cytoskeleton-associated proteins.	Do not rely on cell-ECM adhesions during migration	Charras et al. Nat Rev Mol Cell Biol. 9(9) 2008. Lorentzen et al. J Cell Sci. 124(Pt 8) 2011. Yamazaki et al. Cancer Sci. 96(7) 2005.
**Actin-Enriched Pseudopodial** 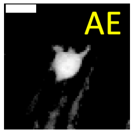	Amoeboidal	Overall rounded cells that have sharp, rather than rounded, protrusions on the membrane	Elevated actin polymerization that drives sharp protrusions. No clear polarization of cytoskeleton and cytoskeleton-associated proteins.	Do not rely on cell-ECM adhesions during migration	Petrie et al. J Cell Sci. 125(Pt 24) 2012. Wyckoff et al. Curr Biol. 16(15) 2006.
**Lamellipodial** 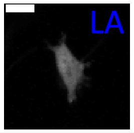	Mesenchymal	Cells with large, flat, fan-like protrusions	Activation of the Arp2/3 complex drives branched nucleation of actin, followed by filament elongation and barbed-end capping. Capping protein is enriched and is necessary to keep filaments short and their number constant. Polarization of Rac1, Cdc42, and PIP3 due to enrichment at the tips of the fan-like protrusions.	Strong cell-ECM adhesions	Abercrombie et al. Exp Cell Res. 59(3) 1970. Petrie et al. J Cell Biol. 197(3) 2012. Mejillano et al. Cell. 118(3) 2004.
**Filopodial** 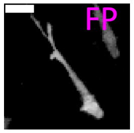	Mesenchymal	Thin, elongated, spindle-like cells	Distinguishable F-actin bundles extending across the polarized cell body supported by elevated actin polymerization and cross-linking by Ena/VASP proteins, along with the activation of Cdc42. Continuous elongation of parallel filaments at their tip is allowed by the prevention of both branching and capping.	Strong cell-ECM adhesions	Nalbant et al. Science. 305(5690) 2004. Trinkaus, J. P. Dev Biol. 30(1) 1973. Mejillano et al. Cell. 118(3) 2004.

Figure S3: Summary of cell shape, signaling and cell-ECM interactions of different migration phenotypes.

S3. Additional Experimental Results

Procedures to compute transition matrices

transition probability between consecutive frames(1 frame = 15 min)

final state \ initial state	BB	AE	LA	FP
BB	0.65	0.34	0.24	0.13
AE	0.10	0.41	0.12	0.06
LA	0.16	0.15	0.52	0.12
FP	0.09	0.10	0.12	0.69
probability	32%	15%	20%	26%

transition rates per hour

transition rates (hr^{-1}) — final state \ initial state	BB	AE	LA	FP
BB		1.35	0.72	0.53
AE	0.39		0.46	0.25
LA	0.62	0.58		0.46
FP	0.35	0.41	0.46	
probability	32%	15%	20%	26%

transition rates (graph representation)

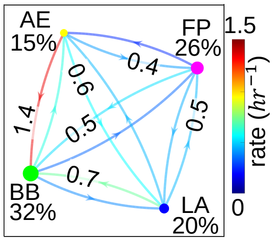

Figure S4: We compute the transition matrix as transition rates, or transition probability per unit time. For instance, Fig. 2C of the main text is obtained by first calculating the transition probabilities between consecutive frames. This leads to a probability matrix where each column sums to 1. We then compute the transition rates, which is the transition probability per hour. Noticing that each frame is 15 min, therefore the transition rates (excluding dwell events) are obtained by multiplying the probability matrix with 4. This leads to transition the matrix we report in this study. To plot Fig. 2C, the rates are color-coded arrows between the nodes.

Transition matrices of additional spheroid samples

regular,1.5 mg/ml

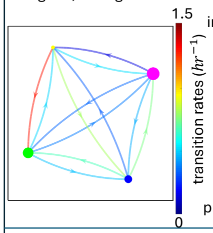

final state \ initial state	BB	AE	LA	FP
BB		1.29	0.67	0.35
AE	0.51		0.34	0.15
LA	0.49	0.81		0.45
FP	0.37	0.53	0.75	
probability	29%	10%	21%	35%

regular,3.0 mg/ml

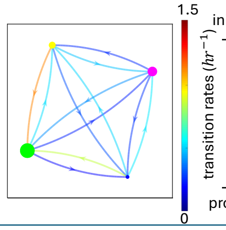

final state \ initial state	BB	AE	LA	FP
BB		1.13	0.86	0.4
AE	0.53		0.51	0.26
LA	0.19	0.31		0.27
FP	0.24	0.48	0.54	
probability	40%	18%	9%	26%

crosslinked,1.5 mg/ml

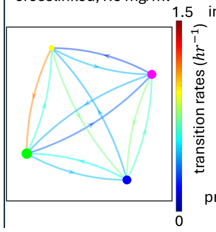

final state \ initial state	BB	AE	LA	FP
BB		1.12	0.58	0.46
AE	0.63		0.46	0.3
LA	0.48	0.74		0.77
FP	0.35	0.46	0.66	
probability	28%	15%	24%	24%

crosslinked,3.0 mg/ml

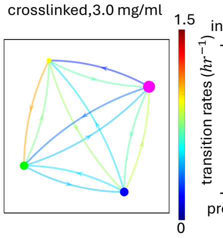

final state \ initial state	BB	AE	LA	FP
BB		1.13	0.86	0.4
AE	0.53		0.51	0.26
LA	0.19	0.31		0.27
FP	0.24	0.48	0.54	
probability	23%	13%	23%	33%

Figure S5: Additional examples of transition matrices calculated by imaging spheroid invasion over 24 hours.

Cell motility exhibit no memory from autocorrelation analysis

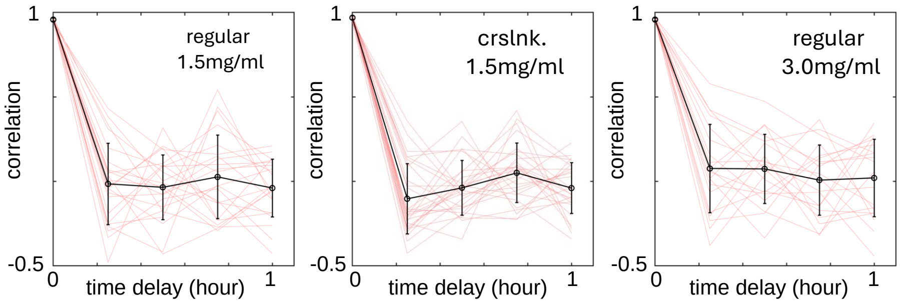

Figure S6: Autocorrelation of velocity shows the motility of cells can be approximately as a Markov process without memory.

Phenotype-dependent average radial positions

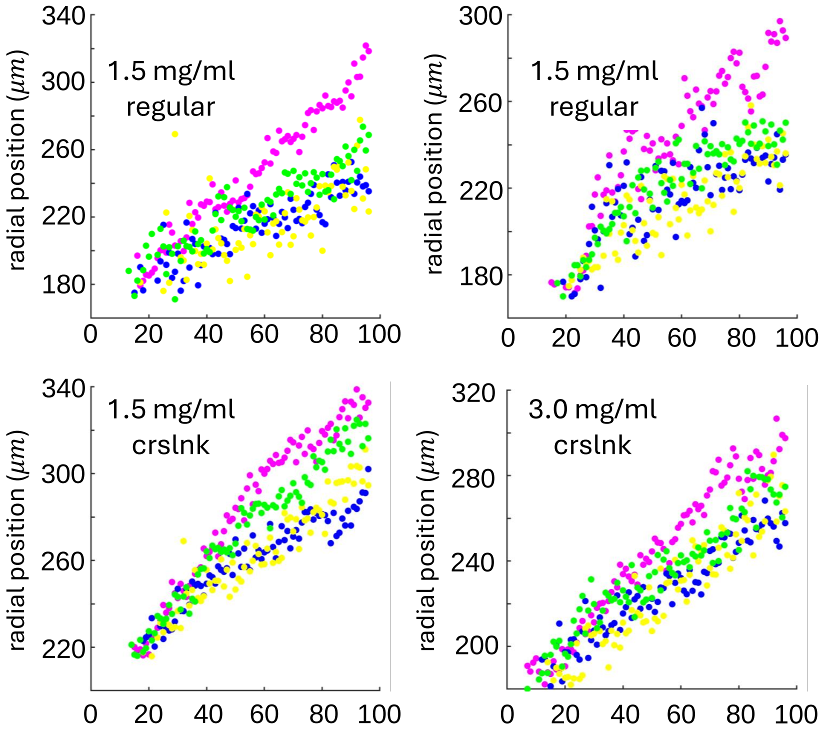

Figure S7: Additional examples show the average radial position of each phenotype, where FP cells exhibit largest invasion depths in all cases.

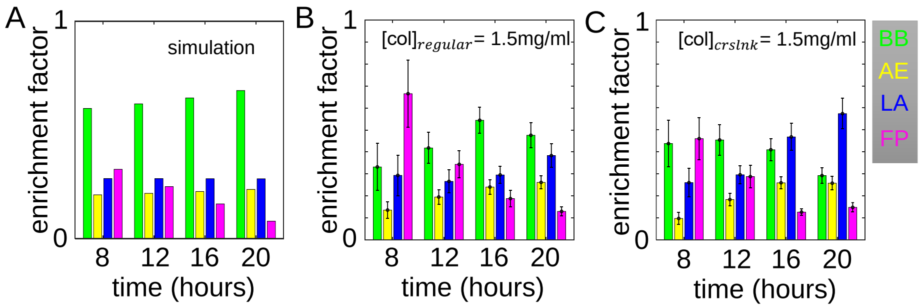

Figure S8: Revised computational model demonstrates the time-dependent phenotype dynamics in agreement with experiments. (A) Simulation results showing the enrichment factors near tumor spheroids. (B-C) The temporal evolution of enrichment factors near tumor spheroids (see main text for definition). The enrichment factor is calculated by sampling events happening within 50 μm of tumor spheroid and within a moving time window of 2 hours span. Bar colors represent enrichment factors for different phenotypes: blebbing (green), actin-enriched pseudopodial (yellow), lamellipodial (blue), filopodial (magenta). Horizontal axis represents the center of the moving window. Error bars are standard deviations obtained from 1000 bootstrap iterations. (B): invasion of MDA-MB-231 cells from tumor spheroids embedded in 1.5 mg/ml regular (not crosslinked) collagen ECM. (C): invasion of MDA-MB-231 cells from tumor spheroids embedded in 1.5 mg/ml photo-crosslinked (abbreviated as crslnk.) collagen ECM.

Time-dependent phenotype transitions near tumor boundary

In order to examine if the adjusted transition rates near the spheroid agree with experimental results, we compute the time-resolved enrichment factor $\eta(t)_X = \frac{\tilde{r}(t)_{X\rightarrow other}}{\tilde{r}(t)_{other\rightarrow X}}$ for each phenotype within 50 μm of tumor boundary. Here X stands for AE, BB, LA, and FP. $\eta(t)_X$ quantifies the bias of cells transition into versus out from phenotype X at time t. We find that the enrichment factor of filopodial cells continuously decrease, while the enrichment factors for other phenotypes remain stable (Fig. S8A). The same trend is observed experimentally, for spheroids in both regular and photo-crosslinked collagen matrices (Fig. S8B-C).

S4. Quantifying the Mechanics and Structure of the ECM

Bulk Rheology Measurements

Bulk Rheology measurements were made on an ElastoSens Bio2 (Rheolution Inc.), which uses new technology to measure the viscoelastic properties of materials using contactless and nondestructive vibrations [7]. The ElastoSens Bio2 comes with specific sample holders that are mounted to a vibration source. This vibration source, paired with the sample holder having a thin, flexible membrane for its bottom, allows the sample to have a small excitation vibration which the machine then measures. By calibrating the machine to the specific sample holders, the machine is able to quantify the viscoelastic properties of the material based on the vibrations induced by the vibration source. The average storage and loss modulus for each of the 8 gel conditions is shown below in Table S1.

**Storage and Loss Modulus**			
Density	Crosslinking	G'	G"
1.5 mg/mL	No	587 Pa	20 Pa
1.5 mg/mL	Yes	555 Pa	103 Pa
3 mg/mL	No	706 Pa	132 Pa
3 mg/mL	Yes	777 Pa	230 Pa
4 mg/mL	No	790 Pa	259 Pa
4 mg/mL	Yes	1137 Pa	383 Pa
6 mg/mL	No	930 Pa	222 Pa
6 mg/mL	Yes	3260 Pa	934 Pa

Table S1: The storage and loss modulus for each of the 8 gel conditions measured on the ElastoSens Bio2.

Figure S9 below shows an example graph of the storage and loss modulus vs time for a 3 mg/mL collagen gel with no crosslinking. To replicate experimental conditions, we first allow the gel to polymerize for 30 minutes in the rheometer at 37°C. Then, the gel is removed from the rheometer and placed under a 365nm UV-light for 5 minutes to induce photocrosslinking. For consistency, all gels are placed under the UV-light regardless of its ability to crosslink. Finally, the gel is placed back into the rheometer to continue measuring the viscoelastic properties of the material for a total of 5 hours. In order to quantify the moduli for a given sample, we look for a 2,000 second window that is relatively consistent in values and take the average value over said window. If the specific sample does not have a clear window, we use a time from a different sample of the same condition to remain consistent across samples. For this specific graph shown in Figure S1, the window chosen was from 9,000 - 11,000 seconds, giving an average value of 721 Pa for the storage modulus, G', and 137 Pa for the loss modulus, G".

Our theoretical model and micromechanical measurements previously all suggest the ECM stiffness can be significantly weakened by cancer cell invasion. To further support the conclusion, we create multispheroid ECM samples and measure the bulk rheology at day 0 an day 1 of invasion. Here the ECM

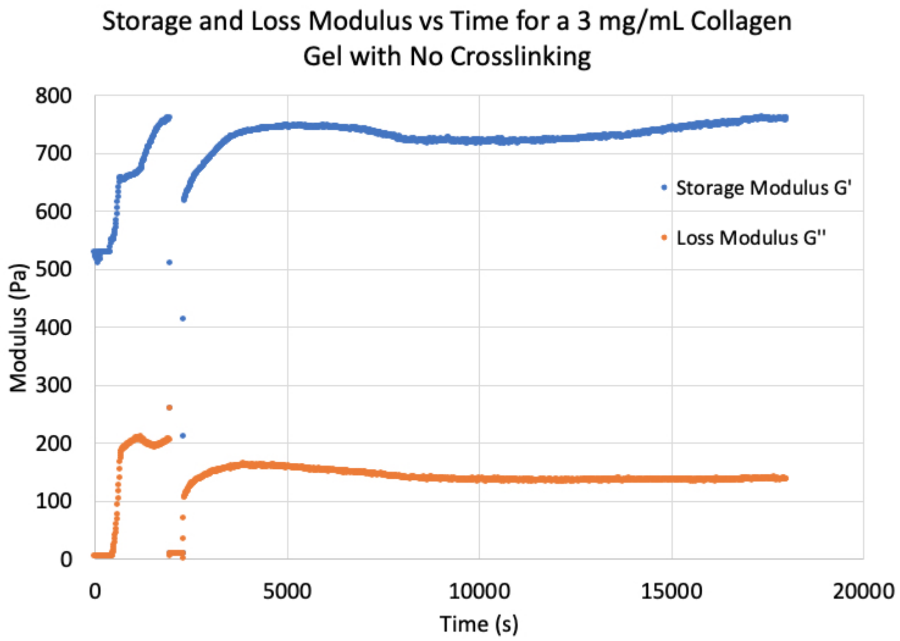

Figure S9: Storage and Loss Modulus vs Time for a 3 mg/mL collagen gel with no crosslinking.

consists of 1.5 mg/ml photo-crosslinked collagen I, and the spheroids have an average distance of 2.4 mm from center to center. Each rheology sample consists 96 spheroids. We find the invasion of spheroids leads to more than 30% reduction in storage modulus, while the stiffness of cell-free sample remains stationary (Fig. S10).

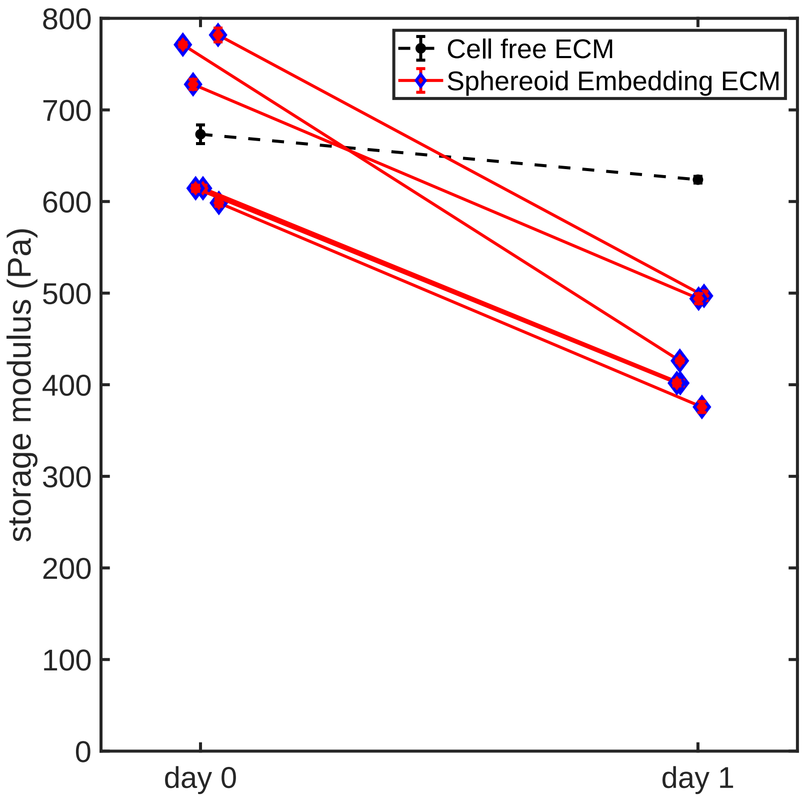

Figure S10: Spheroid invasion reduces storage modulus of surrounding ECM. Each line represents one sample. Errorbars show mean±std from multiple measurements of a sample.

Pore Size Quantification

Figure S11 below shows a confocal reflection image of a 1.5 mg/mL regular collagen gel compared to a 1.5 mg/mL crosslinked collagen gel. In order to quantify the structure of the porous collagen matrix, we fit circles to the blank space between fibers to calculate the pore size. To do this, we first binarize the image and then take a small rolling ball radius of 3 pixels to despeckle the image for any noise. Figure S12 below shows the outcome image after binarizing and despeckling the raw confocal image for both the same 1.5 mg/mL regular collagen gel and 1.5 mg/mL crosslinked collagen gel. Now that we have a binarized image that best represents the fiber structure, we compute the distance transform of the image to calculate the distance between bright pixels that represents the distance between fibers. Since this distance transform is for all pixels, we are left with a distribution of the pore size. The distribution is modeled with a Lognormal to better fit our data rather than simply taking the average value. Figure S13 below shows the distribution of the pore size along with the Lognormal fit of the distribution for both the same 1.5 mg/mL regular collagen gel and 1.5 mg/mL crosslinked collagen gel. For these specific images, the average value pore size radius given by the Lognormal fit is 10.47 pixels and 10.90 pixels, or 2.81 μm and 2.93 μm, respectively, using our lens' pixel to micron ratio of 3.72.

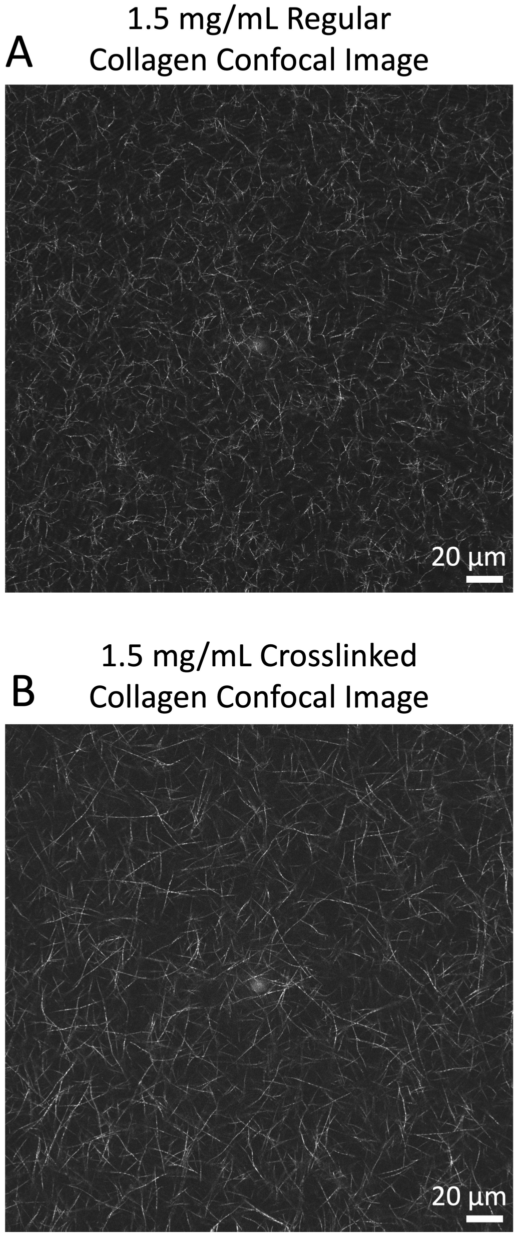

Figure S11: Confocal reflection images of typical collagen gels. Scale bars are 20 μm. (A): 1.5 mg/mL regular collagen gel. (B): 1.5 mg/mL crosslinked collagen gel.

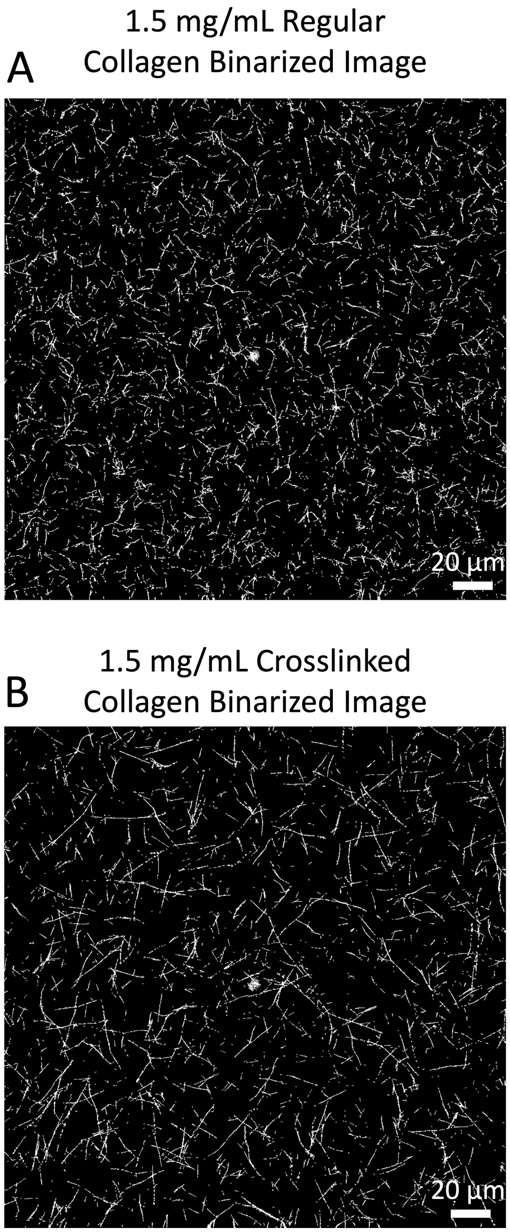

Figure S12: Binarized and despeckled images of typical collagen gels. Scale bars are 20 μm. (A): 1.5 mg/mL regular collagen gel. (B): 1.5 mg/mL crosslinked collagen gel.

A

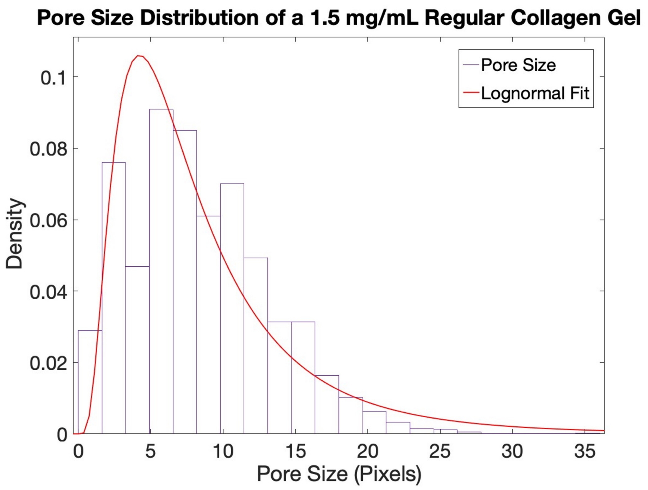

B

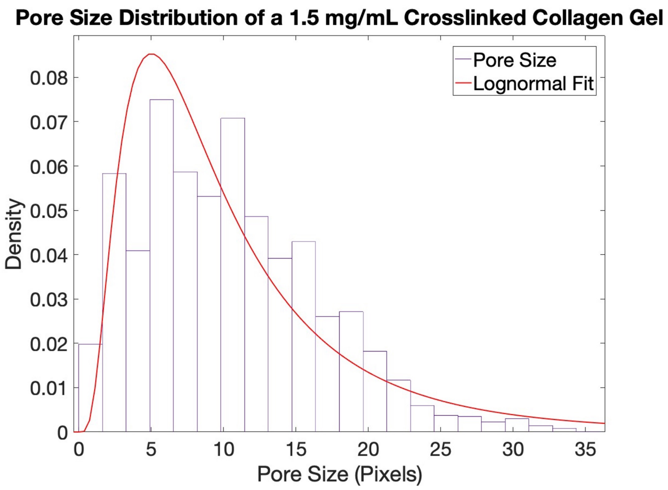

Figure S13: Pore size distributions of typical collagen gels along with their Lognormal fit. (A): Pore size distribution of a 1.5 mg/mL regular collagen gel. The Lognormal fit gives an average pore size radius of 10.47 pixels, or 2.81 μm, using our lens' pixel to micron ratio of 3.72. (B): Pore size distribution of a 1.5 mg/mL crosslinked collagen gel. The Lognormal fit gives an average pore size radius of 10.90 pixels, or 2.93 μm, using our lens' pixel to micron ratio of 3.72.

S5. Additional details of computational modeling

The following parameters are used to produce the result presented in the main text, Fig. 5.

Rates (h^{-1})	AE	BB	FP	LA
AE	0	1.35	0.41	0.58
BB	0.62	0	0.35	0.39
FP	0.25	0.53	0	0.46
LA	0.46	0.72	0.46	0
v (μm/h)	11.2	6.8	18.0	7.2

Table S2: Transition rates and cell speeds used in the model

We have verified that adding a diffusion constant to the model equations (see Eq. 1 in the main text) does not qualitatively affect the results. This is shown in Fig. S14, where we plot the invasion profiles of the different phenotypes as a function of time. Here, the diffusion constant was taken to be identical for all phenotypes and equal to $D = 20\mu^2/\text{h}$, consistent with the stepsizes reported in Fig. 2. As in Fig. 5 of the main text, phenotype FP displays a larger invasion depth.

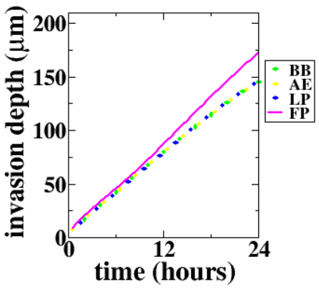

Figure S14: Invasion profiles of each phenotype from the computational model with an added diffusion constant. As in the main text, the transition rates from phenotype FP close to the spheroid are linearly decreased over time (see Eqns. 3).

References

[1] C. Z. Eddy, H. Raposo, A. Manchanda, R. Wong, F. Li, and B. Sun, "Morphodynamics facilitate cancer cells to navigate 3d extracellular matrix," *Scientific Reports*, vol. 11, no. 20434, 2021.

[2] J. C. CROCKER and D. G. GRIER, "Methods of digital video microscopy for colloidal studies," *JOURNAL OF COLLOID AND INTERFACE SCIENCE*, vol. 179, no. 0217, 298â310, 1996.

[3] C. Z. Eddy, A. Naylor, C. T. Cunningham, and B. Sun, "Facilitating cell segmentation with the projection-enhancement network," *Physical Biology*, vol. 20, no. 6, 2023.

[4] C. Stringer, T. Wang, M. Michaelos, and M. Pachitariu, "Cellpose: A generalist algorithm for cellular segmentation," *Nature Methods*, vol. 18, pp. 100–106, 2021.

[5] C. Cortes and V. N. Vapnik, "Support-vector networks," *Machine Learning*, vol. 20, pp. 273–297, 1995.

[6] A. Ben-Hur, D. Horn, H. T. Siegelmann, and V. Vapnik, "Support vector clustering," *Journal of Machine Learning Research*, vol. 2, pp. 125–137, 2001.

[7] C. Ceccaldi *et al.*, "Validation and application of a nondestructive and contactless method for rheological evaluation of biomaterials," *Journal of Biomedical Materials Research. Part B, Applied Biomaterials*, vol. 105, no. 8, pp. 2565–2573, 2017.
